# Supplementary material for: Amelioration of Cyclosporine A-Induced Acute Nephrotoxicity by Cordyceps cicadae Mycelia via Mg+2 Reabsorption and the Inhibition of GRP78-IRE1-CHOP Pathway: In Vivo and In Vitro
Source: Int J Mol Sci. 2023 Jan 1;24(1):772. doi: 10.3390/ijms24010772 (PMC9820889; doi:10.3390/ijms24010772)
Supplement: Supplementary file 1 [file ijms-24-00772-s001.zip › ijms-2081897-supplementary.pdf]

Supplementary material

Cordyceps cicadae mycelium against cyclosporine-induced acute nephrotoxicity via the regulation of renal TRPM 6 and 7 channel activity in rats

Zong-Han Wu[1], Chun-Hung Chiu[1,2], Chin-Chu Chen[3], Charng-Cherng Chyau[1,*], Chi-Hung Cheng[1,4,*]

[1] Research Institute of Biotechnology, Hungkuang University, Taichung 43302, Taiwan

[2] Department of Program in Animal Healthcare, Hungkuang University, Shalu District, Taichung City 43302, Taiwan; chchiu@hk.edu.tw

[3] Grape King Biotechnology Center, Chung-Li City, Taoyuan County 320, Taiwan

[4] Department of Nephrology, Catholic Mercy Hospital, Hukou Township, Hsinchu County, Taiwan; chc.ede@msa.hinet.net

* Correspondence: ccchyau@hk.edu.tw (C.-C.C.); chc.ede@msa.hinet.net (C.-H.C.); Tel: +886-26318652; Fax: +886-4-26525386

Table S1. The sequence of primers used in this study.

Name	Sequence(5′ to 3′)
IL-1β -forward	*ccagggacaggatatggagca*
IL-1β - reverse	*ttcaacacgcaggacaggtacag*
IRE1α- forward	*gatcctcagaaacgcccctc*
IRE1α- reverse	*tctgtcgctcacgtcctg*
PERK- forward	*aggacagaggggacagagttg*
PERK- reverse	*taatgaccttttcttccctgctcc*
ATF6- forward	*agtattttgtccgcctgccg*
ATF6- reverse	*gcagaatcccaatcttcatcca*
CHOP(GADD153)- forward	*cagagctggaacctgaggag*
CHOP(GADD153)- reverse	*tggatcagtctggaaaagca*
